# Differences in Collaboration Patterns across Discipline, Career Stage, and Gender

**DOI:** 10.1371/journal.pbio.1002573

**Published:** 2016-11-04

**Authors:** Xiao Han T. Zeng, Jordi Duch, Marta Sales-Pardo, João A. G. Moreira, Filippo Radicchi, Haroldo V. Ribeiro, Teresa K. Woodruff, Luís A. Nunes Amaral

**Affiliations:** 1 Department of Chemical and Biological Engineering, Northwestern University, Evanston, Illinois, United States of America; 2 Department d’Enginyeria Informàtica i Matemàtiques, Universitat Rovira i Virgili, Tarragona, Spain; 3 Department d’Enginyeria Química, Universitat Rovira i Virgili, Tarragona, Spain; 4 Center for Complex Networks and Systems Research, School of Informatics and Computing, Indiana University, Bloomington, Indiana, United States of America; 5 Departamento Fisica, Universidade Estadual de Maringá, Maringá, Parana, Brazil; 6 Department of Obstetrics & Gynecology, Feinberg School of Medicine, Northwestern University, Chicago, Illinois, United States of America; 7 Institute for Women’s Health Research, Northwestern University, Chicago, Illinois, United States of America; 8 Department of Physics & Astronomy, Northwestern University, Evanston, Illinois, United States of America; 9 Northwestern Institute on Complex Systems, Northwestern University, Evanston, Illinois, United States of America; University of California San Francisco, UNITED STATES

## Abstract

Collaboration plays an increasingly important role in promoting research productivity and impact. What remains unclear is whether female and male researchers in science, technology, engineering, and mathematical (STEM) disciplines differ in their collaboration propensity. Here, we report on an empirical analysis of the complete publication records of 3,980 faculty members in six STEM disciplines at select U.S. research universities. We find that female faculty have significantly fewer distinct co-authors over their careers than males, but that this difference can be fully accounted for by females’ lower publication rate and shorter career lengths. Next, we find that female scientists have a lower probability of repeating previous co-authors than males, an intriguing result because prior research shows that teams involving new collaborations produce work with higher impact. Finally, we find evidence for gender segregation in some sub-disciplines in molecular biology, in particular in genomics where we find female faculty to be clearly under-represented.

## Abstract 2

An empirical analysis of researchers’ publications reveals that females have fewer distinct coauthors yet have a lower chance of repeating previous coauthors than their male counterparts.

## Author Summary

Collaboration plays an increasingly important role in promoting research productivity and impact. What remains unclear is whether female and male researchers differ in their collaboration practices. In our study, we report on an empirical analysis of the complete publication records of 3,980 faculty members in six science, technology, engineering, and mathematical disciplines at select U.S. research universities. First we found that female faculty have significantly fewer distinct co-authors over their careers than males, but that this difference can be fully accounted for by females’ lower publication rate and shorter career lengths. Next, we find that female scientists have a lower probability of repeating previous co-authors than males, an intriguing result because prior research shows that teams involving new collaborations produce work with higher impact. Finally, we find evidence for gender segregation in some sub-disciplines in molecular biology, in particular in genomics where we find female faculty to be clearly under-represented.

## Introduction

It is widely acknowledged that collaboration is critical to the scientific enterprise [1–7]. Although the motivations determining collaboration propensity is still the subject of much research, scientists benefit from collaboration both in terms of productivity and impact [8–12]. For example, Bordons et al. [13] showed that for biomedical research there is a positive correlation between productivity and collaboration at the author level, and Wuchty et al. [14] showed that teams produce publications with higher impact than individuals. Moreover, teams that include novel collaborations have a greater likelihood of producing higher impact work [15, 16].

Since research suggests that collaboration patterns affect a researcher’s career performance, it is important to understand whether there are gender differences in collaboration patterns [17, 18]. Indeed, Kyvik and Teigen [19] reported that the productivity of both genders is positively correlated with the level of collaboration, and that females have fewer single-author works than males.

Prior research suggests that women tend to be more collaborative and less competitive than men in decision making, making them potentially better collaborators [20–22], but recent studies have reported contradicting results about which gender is more collaborative [23–27].

Because most STEM fields have much larger numbers of males than of females, homophily would suggest that female academics have fewer opportunities for collaboration [28]. McDowell et al. [29] find evidence of gender homophily in collaborator choice among a sample of economists and that females preferentially apply to larger departments to increase their chances of finding collaborators. Bozeman et al. not only find evidence of the same gender homophily [24] but also that, after controlling for gender disparities, females overall collaborate more than males [26].

To investigate the role of gender in collaborative behavior, we perform a large-scale empirical analysis on the publication records of faculty members for six STEM disciplines. Our analyses yield three main findings. First, female faculty have significantly fewer distinct co-authors than male faculty, but that this difference can be fully accounted for by the shorter career lengths of current female faculty and their lower publication rate. Second, female faculty tend to have a lower probability of repeating a collaboration, a strategy that has been shown to produce work of greater impact. Third, for the discipline of molecular biology, we find evidence for gender segregation in some sub-disciplines. In particular, we find that female faculty are clearly under-represented in genomics.

### Data

We obtain complete faculty rosters, as of Fall 2010, for departments of chemical engineering, chemistry, ecology, materials science, molecular biology and psychology from several top research universities in the United States (US) (S1 and S2 Tables). We consider all active faculty members as of 2010, including tenure-track and research faculty, but exclude emeritus professors. We identify the researchers’ gender from their departmental website photograph. If they have no photograph we use their given name to identify the gender (faculty with ambiguous names were excluded). We then obtain bibliometric data for 3,980 faculty members from Thomson Reuters’ Web of Science (WoS) based on the biographical information listed on their websites and *curricula vitae*. See [30] for details on data acquisition and validation, and Table 1 for aggregate statistics.

**Table 1:** Characteristics of the faculty cohorts in our study.

Discipline	Depts.	Faculty			Publications		
		Female	Male	Ratio	Female	Male	Ratio
Chemical Engineering	31	98	567	1:5.8	6,392	66,328	1:10.4
Chemistry	35	198	1,020	1:5.2	13,790	137,723	1:10.0
Ecology	15	106	328	1:3.1	3,976	22,425	1:5.6
Materials Science	26	98	473	1:4.8	9,538	75,373	1:7.9
Molecular Biology	11	168	474	1:2.8	9,882	51,234	1:5.2
Psychology	10	171	279	1:1.6	7,143	20,976	1:2.9
**Total**	129	839	3,141	1:3.7	50,721	374,059	1:7.4

## Results

### Gender differences in number of collaborators

Since scientific publications are the direct product of scientific research and collaboration, the number of distinct co-authors a researcher has accrued throughout her career is a good proxy of how strongly she seeks collaborations. Because collaboration patterns may be discipline-specific, we examine each discipline separately [31]. Moreover, because collaboration patterns may depend on career stage, we also account for career stage in our analyses.

We focus on the number of *distinct* co-authors; that is, we count only once co-authors that appear multiple times in the publications of an individual. We do this because co-authoring publications with new collaborators more likely indicates the introduction of new expertise into the team and the expansion of one’s professional network.

**Fig 1:**
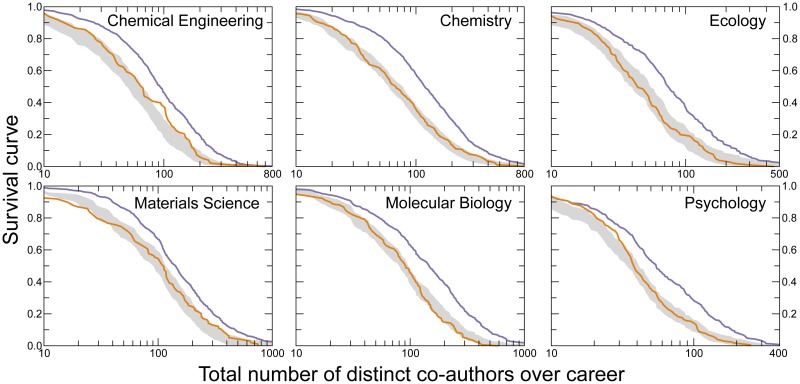
Lower number of publications by female scientists results in lower total number of distinct co-authors. Survival curve of the total number of co-authors over careers of females (orange) and males (purple). We test the null hypothesis that there is no gender difference in the total number of distinct co-authors for females and males with similar number of publications. The grey shaded region indicates the 95% confidence interval obtained under the null hypothesis. To construct the confidence interval, we generate samples of *N*_*F*_ males, where *N*_*F*_ is the number of females in our dataset. For a female with *n*_*F*_ publications, we select a male whose number of publications falls in the range of [0.8 *n*_*F*_, 1.2 *n*_*F*_] (see Materials and Methods). Note that the curve for females falls inside the confidence interval, indicating that after correcting for number of publications, females and males have comparable numbers of distinct co-authors over their careers. The curve for males falls outside the confidence interval because some male researchers in the dataset have very large numbers of publications (see Fig 7 of [30]). Data for this figure are in S1 Data.

We calculate the distribution of total number of distinct co-authors over the career of the scientists in our database. Our raw results show that for all six disciplines, females on average have a significantly lower number of distinct co-authors over their careers than males (Fig 1). However, in order to properly interpret these results, we must account for the fact that until 1980 there were hardly any female faculty, which implies that female faculty typically have shorter career length and thus are likely to have fewer publications than their male colleagues [30]. Moreover, because of the gender gap in the number of publications [30, 32], it is necessary to control for publication rate when comparing the number of co-authors of females and males. Thus, we test the null hypothesis that there is no gender difference in the number of distinct co-authors when controlling for the number of publications (see Materials and Methods). The confidence intervals constructed under this hypothesis show that once we account for the number of publications, the observed difference in the distribution of the number of distinct co-authors of female and male faculty is not statistically significant (Fig 1).

### Repeated co-authors and propensity to collaborate

The data from Fig 1 shows that female and male faculty accrue an average number of new distinct co-authors per publication that is indistinguishable from the average for males. However, this observation does not imply that females and males accrue new collaborators in the same manner, or that they have the same propensity to collaborate.

#### Accruing new collaborators

Consider a publication of researcher *i* and *n*_*c*_ co-authors. The number *n*_*n*_ of distinct co-authors that *i* accrues can be expressed as

$$n_{n}=n_{c}\;\left(1-f_{r}\right),$$ (1)

where *f*_*r*_ is the fraction of repeated co-authors. Eq (1) makes explicit that both team size (that is, *n*_*c*_) and propensity to repeat collaborations affect the number of new distinct co-authors to be gained from each publication. We first investigate the effect of the repetition of co-authors on the gender disparity in the number of distinct co-authors. Researchers who frequently co-author with the same team will not accumulate co-authors as rapidly as those who seek out new collaboration opportunities. To quantify the tendency to repeat previous co-authors, we calculate *f*_*r*_ for each author, and obtain the distribution of *f*_*r*_ for both genders for each discipline. We then test whether the two samples could have been drawn from the same distribution.

**Fig 2:**
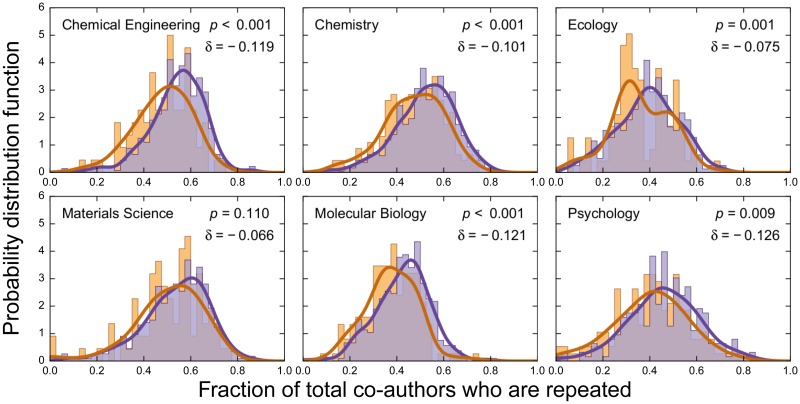
Gender differences in the propensity to co-author with prior collaborators. Probability distribution of the fraction of total coauthors who are repeated for all females (orange) and males (purple) in the dataset with at least 10 publications. We exclude single-author publications. Orange and purple lines are kernel density estimation of the distributions for females and males with bandwidth given by Scott’s Rule [34]. We obtain *p*-values for the validity of the null hypothesis that the samples were drawn from the same distribution using the Kolmogorov-Smirnov test. For all disciplines, we find $\delta =2\left({\bar{f}}_{r,F}-{\bar{f}}_{r,M}\right)/\left({\bar{f}}_{r,F}+{\bar{f}}_{r,M}\right)<0$, where ${\bar{f}}_{r,F}$ and ${\bar{f}}_{r,M}$ are the average *f*_*r*_ of the female and male faculty, respectively. Females have *f*_*r*_ smaller than those of males, suggesting that, except for materials science, female faculty have a lower propensity than male faculty to repeat collaborations. Data for this figure are in S2 Data.

We show in Fig 2 the probability distribution functions of *f*_*r*_ for females and males. The data show that females have an *f*_*r*_ approximately 20% smaller than males, indicating that female faculty repeat co-authors less frequently than male faculty. More frequent repetition of co-authors may also be an indicator that a few co-authors are responsible for most collaborations. We use the Gini coefficient [33] and the disparity index to quantify the degree of inequality in the distribution of collaboration frequencies, and find that females do tend to distribute their co-authoring opportunities more equally among their collaborators than males (S1, S2 and S3 Figs).Although the gender difference in the tendency to repeat co-authors is significant, our ability to establish its statistical significance on the total number of distinct co-authors is hampered by the heterogeneity in team size and number of publications (S4 Fig).

#### Average team size

**Fig 3:**
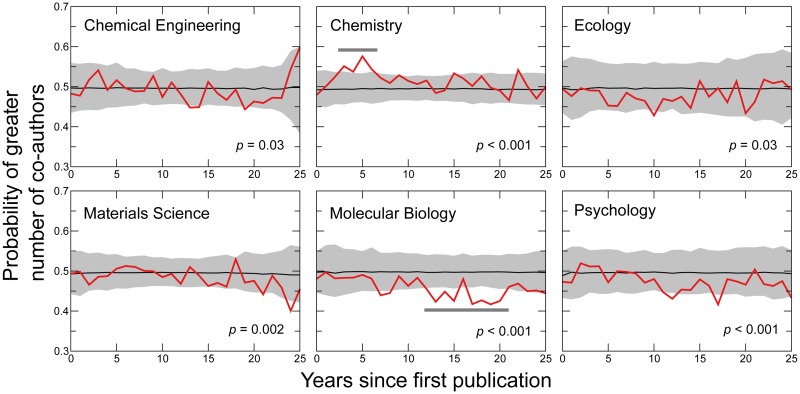
Male and female faculty have similar number of co-authors per publication for five other disciplines, but not for molecular biology. Probability of females having greater number of co-authors per publication in a given year of her career than a male peer at the same career stage (red lines). We use z-scores to account for the increasing size of research teams and the fluctuations over career stage (see Materials and Methods). We indicate the 99% confidence intervals by the grey areas, and the medians of the probabilities obtained from random ensembles by black lines. The p-values are obtained under under the null hypothesis that there is a 99% probability of any value being outside the confidence interval. Note that although the difference in the average size of teams appears to be statistically significant, it is not consistent along the career stage, except for chemistry for the first few years, and for molecular biology in later career stages (dark horizontal bars). Data for this figure are in S3 Data.

We next study the average number of co-authors per publication, *n*_*c*_. Researchers who collaborate with larger teams have higher numbers of co-authors per publication. However, the number of co-authors changes as a function of the publication year and author’s career stage (S5 Fig). Since female faculty entered academia more recently and on average have shorter career lengths than male faculty [30], we need to account for these two factors when comparing team sizes. In Fig 3 we show that, except for molecular biology, the two genders do not differ significantly in the number of co-authors per publication when their publication year and career stage are taken into consideration.

### The case of molecular biology

Our findings for molecular biology are intriguing. While there are no significant differences during the first ten years, beyond ten years, publications authored by females in molecular biology have significantly lower number of co-authors per publication than those authored by males. To further detail this observation, we bin the publications authored by females according to the number of co-authors, after accounting for increases in team size over the period considered. Assuming that females do not prefer any particular team size, the fraction of publications by females in each bin should remain approximately constant. For each bin, we then calculate how much the observed number of publications by females deviate from the number expected from the null hypothesis using the hypergeometric distribution (see Materials and Methods). S6 Fig demonstrates that female faculty in molecular biology departments have a distinct behavior from females in other disciplines: They consistently author significantly more publications than expected in teams smaller than average, and significantly fewer publications than expected in teams larger than average. We make this fact visually apparent by shading in grey regions where the observed value is significantly different from the null hypothesis.

#### Segregation among sub-disciplines

Although we restrict our analysis to researchers within the same discipline, academic disciplines such as molecular biology comprise several sub-disciplines. If females and males are segregated across sub-disciplines so that more males work in sub-disciplines with large teams, and more females in those with small teams, then this segregation could give rise to the gender gap in the average number of co-authors per publication.

**Fig 4:**
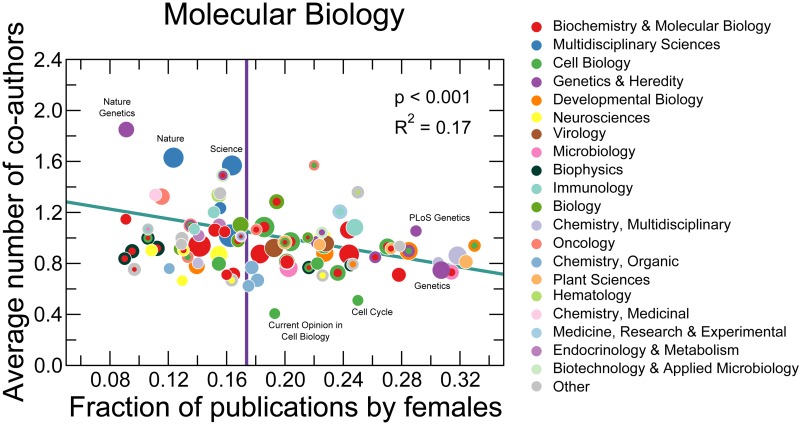
Female faculty in molecular biology departments publish more in journals and sub-disciplines where typical team size is smaller. We show correlation between the average number of co-authors corrected for the annual average versus the fraction of publications authored by females, grouped by journal. We only consider publications authored after the tenth year mark in an author’s career. We restricted the publication types to “article”, “letter”, and “note.” The size of the circle is proportional to the logarithm of the number of publications in that journal or sub-discipline. We use journal category in the *ISI Journal Citation Report* as the sub-disciplines. Journals with multiple categories are plotted as concentric rings. The purple line indicates the total average fraction of publications by females for all the publications authored by faculty in molecular biology in our cohort, *f*_*M*_ (17.3%). The blue line is a weighted linear regression, in which we assign to each journal a weight equal to the number of publications. We only include data points within the range of [0.5*f*_*M*_, 2*f*_*M*_]. Data for this figure are in S4 Data.

We find that at journal level the average number of co-authors is strongly and significantly anti-correlated with the fraction of publications authored by females (Fig 4). The strong and statistically significant anti-correlation indicates that females publish more in journals (and, presumably, sub-disciplines) where the typical team size is smaller, and less in those where the typical team size is larger (see S7 Fig through S11 Fig for results for other disciplines).

The journal-level analysis strongly suggests the existence of gender segregation across sub-disciplines. However, many journals are multi-topic and even multidisciplinary, thus they may not accurately represent narrower research topics. To overcome this limitation of the journal-level analysis, we must determine the research topic of each publication at a finer scale. To this end, we use a highly accurate and reproducible topic classification algorithm to identify the topics of publications [35]. We identify a total of 69 topics using the titles and abstracts from the set of 61,116 publications by molecular biology faculty in our database. S3 Table lists the identified topics and the most representative words and journals associated with them.

**Fig 5:**
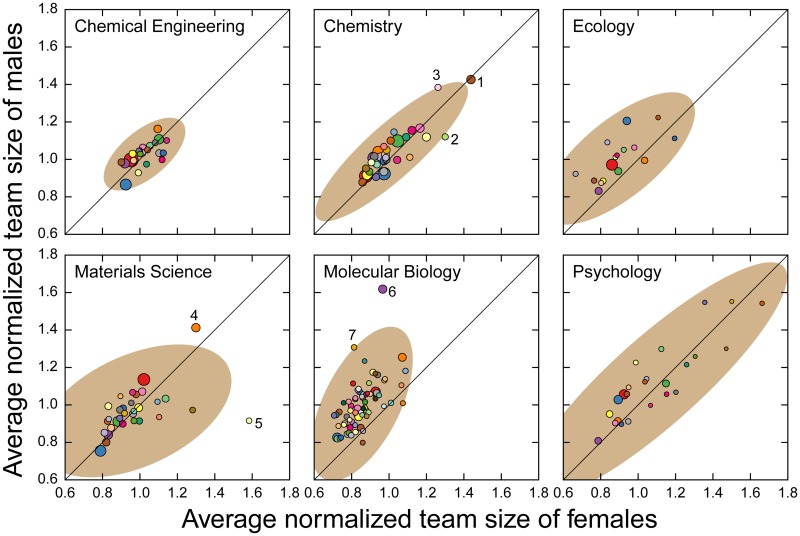
Topic dependence of female representation in publications in the six disciplines. We show the average number of co-authors corrected for the annual average for male faculty versus that for female faculty. Note for molecular biology most of the data points fall above the line *y* = *x*, indicating that for most topics females work in smaller teams than males. We label the seven topics which fall outside the 99% confidence region (brown ellipse) (see Table 2 for topic details). Data for this figure are in S5 Data.

For the publications in each topic, we calculate the average team size and fraction of publications by females (Fig 5). Using a 99% confidence region [36], we identify seven topics that are outliers; of those, two are in molecular biology (Table 2). All the outlier topics in chemistry and of the outlier topics in materials science actually have larger representations of publications by female faculty and larger team sizes. In contrast, the outlier topics in molecular biology have just larger team sizes. Looking at the representative journals for each of the outlier molecular biology topics, it becomes clear that topic 6 refers to genomics.

**Table 2:** Topics within considered disciplines that are outliers when considering the differences in average team size between male and female faculty in our database.

Discipline	Topic	Outlier topic	Representative journals	No. publs.	Normalized ratio by females	Mean normalized team size
Chemistry	C4	1	Cancer Research, Bioconjugate Chemistry, Antimicrobial Agents and Chemotherapy	4,809	1.5	1.43
	C14	2	Nucleic Acids Research, Physical Review E, Genome Biology	1,354	1.3	1.37
	C18	3	Journal of Membrane Science, Radiochimica Acta, Journal of Natural Products	1,399	1.2	1.14
Materials Science	M0	4	Biomaterials, PNAS, Journal of Biological Chemistry	4,547	2.0	1.39
	M29	5	Organome tallics, Journal of Chemical Physics, Surface Science	1,742	1.0	0.99
Molecular Biology	B5	6	Nature Genetics, Genetics, Nucleic Acids Research	4,186	1.0	1.51
	B10	7	Molecular Biology and Evolution, Genetics, American Journal of Botany	899	1.1	1.22

**Topic** represents the topic number identified by the topic classification algorithm and is field-specific [35];**Outlier topic** represents the topic in Fig 5.

Genomics (topic B5) is particularly relevant when attempting to explain the smaller team sizes of female authored molecular biology papers. Genomics is unique because it has a very striking under-representation of females and markedly larger team sizes. Moreover, because it is a topic with a very large number of publications, it strongly affects the characteristics of the entire discipline. These results prompt the question of why females are under-represented in genomics. S4 Table shows that 19 of the 20 most prolific researchers in our database working in genomics are male. A recent study suggests that the labs of prominent male researchers have lower than average fractions of female graduate students and postdocs [37]. Since the protégés of prominent scientists have such an important role in populating faculty positions in molecular biology, the under-representation of females in those labs propagates all the way to the level of tenured faculty.

In order to investigate the origins of the distinct characteristics of the outlier topics, we turn again to the lists of the scientists with the most publications in each topic (S4 and S5 Tables). We then repeat the analysis of Fig 5 but excluding the publications of the 5 most prolific scientists for each outlier topic. Strikingly, we find that the characteristics of these topics revert to the mean for the entire discipline. That is, the gender of the most prolific authors determines the characteristics of the topic. We believe that this finding raises an important question: Why females have not been able to succeed in genomics in proportion to their numbers? No female in our dataset made it into the top 10 most prolific scientists in genomics, the first female appearing in 12*^th^* place. If genomics was gender blind, and considering that females comprise 26% of the biology researchers in our database, this would be an unlikely situation (*p* ≃ 0.0095).

## Discussion

A number of recent studies support the hypothesis that there are gender differences in collaboration patterns [17, 18] and that collaboration has a significant impact on scientific productivity and impact [14, 15]. Evidence suggests that self-selection among female researchers due to greater career risks, and female scientists’ decreased access to funding can, respectively, cause gender differences in publication rate and impact [29, 30].

Our present analysis conclusively shows that females do have fewer distinct co-authors over their careers, but that this gap can be accounted for by differences in number of publications. We also find evidence for the hypothesis that female scientists are more open to novel collaborations than their male counterparts, a behavior that was shown to correlate with producing work of greater impact [15].

It could be, however, that females have fewer distinct collaborators not purely because, as the females in our cohort they publish fewer publications, but because female scientists do not participate in research teams to the same extent as male scientists. We believe that this possibility is unlikely since there is strong evidence that females are generally more collaborative than males both in academic life [26, 27] and in other realms [20–22].

Concerning our finding that females appear to be more likely to engage new collaborators, it could be that females are simply more effective collaborators and are able to make the most of their lower representation in STEM disciplines. Wolley et al. showed that females typically have greater group intelligence than males [38] giving some credence to this hypothesis. An alternative explanation for the greater repetition of collaborations by males is unwarranted authorship in publications for the purpose of increasing one’s publication counts. Anecdotal evidence suggests that, while the number of scientists pursuing such gaming of the system is small, they do tend to be male.

Lastly, our finding of female exclusion from genomics is of particular interest, especially because of what it may imply concerning the cultural milieu of this sub-discipline. The importance of culture on gender segregation is supported by recent studies showing the existence of gender stereotyping in physics and its negative consequences for females in that field [39, 40]. It is known that in some molecular biology sub-disciplines such as telomere research (topic B21) the participation of female scientists has been encouraged. Indeed, 6 of the 10 most prolific researchers in this topic are female (S6 Table). The top three researchers, Elizabeth Blackburn, Virginia Zakian, and Carol Greider conducted their doctoral research under the mentorship of Joseph Gall, who is known for having supported female scientists at a time when misogyny was widely accepted. The important role of prominent scientists in encouraging both males and females to pursue careers in research is also illustrated by William H Bragg’s role in the recruitment of female scientists to crystallography. In contrast, the cultural milieu in institutions such as Genentech [41] likely had a chilling effect on female participation in genomics.

One caveat of our study is that it is limited by the fact that we are only able to track those scientists that persisted within academia. We believe it is important to also investigate to what extent our findings would still hold for scientists that were unable to remain in academic positions at top universities. In a perverse way, it could be that females’ propensity to collaborate creates both better publications and a successful research program, and greater risk when the time comes for tenure decisions. Another caveat is that we are not able to identify which coauthors may be trainees (graduate students or post-docs), a situation that in many cases would be more representative of mentorship than of typical collaboration.

## Materials and Methods

### Co-author names matching

To calculate the number of distinct co-authors for a researcher, we used the following procedure. For each researcher, we maintain a set of standardized co-author names. For each co-author name, we convert the name to a string of last name and first name initials. For example, a co-author named “Jane Linda Smith” will be converted to “Smith JL”. For each publication, we standardize the names of the co-authors, and add them to the set. We finally count the number of elements in the set.

Note that using this procedure, we treat “Jane Linda Smith” and “Jane Lily Smith” as the same name, because they are both converted to the string “Smith JL”. Also, we treat “Jane Linda Smith” and “Jane Smith” as different names, since the former is converted to “Smith JL”, while the latter is converted to “Smith J”. In reality, for a single author’s co-authors, the probability for either case to happen is very small, hence the error rate of our procedure is very low.

### Confidence interval for the survival curve of total number of distinct co-authors

We use matched sampling to obtain the confidence interval for the survival curve of total number of distinct co-authors. We consider the null hypothesis that there is no difference in the total number of co-authors between females and the males with similar number of publications. To construct the confidence interval, we generate samples of *N*_*F*_ males, where *N*_*F*_ the number of females in our dataset. For a female with *n*_*F*_ publications, we select a male whose number of publications falls in the range of [0.8 *n*_*F*_, 1.2 *n*_*F*_], a range small enough to produce good matches but large enough that there is at least one match. We then compute the survival curve for the obtained sample of male authors. We obtain the confidence interval by repeating this procedure 1,000 times.

The procedure is similar for the null hypothesis that there is no difference in the total number of co-authors between females and the males with equal number of publications, except that the sample of males consists of males who have the same number of publications as the females.

### Measuring gender difference in the distribution of collaboration opportunities

We use two methods, the Gini coefficient and the disparity index, to measure how homogeneously each author distributes all her/his collaboration opportunities among her/his co-authors. A high Gini coefficient or disparity index indicates inhomogeneity of collaboration frequency distribution, where the author collaborates highly frequently with only a small portion of her/his co-authors, but only a few times with each of the remaining majority. Thus, this author has a high propensity to concentrate her/his collaboration opportunities on a few co-authors. A low Gini coefficient or disparity index indicates that the author collaborates with each of her/his co-authors about equally frequently.

*Gini coefficient*. Consider author *a* with *n*_*c*_ co-authors. For each co-author *c*_*i*_ of *a*, we count the times of collaboration between *a* and *c*_*i*_, *y*_*i*_. That is, the number of publications *a* has co-authored with *c*_*i*_. We next arrange *y*_*i*_ in non-decreasing order, where *y*_*i*_ ≤ *y*_*i*+1_. The Gini coefficient of author *a* is calculated as

$$G\left(a\right)=\frac{2\sum_{i=1}^{n_{c}}iy_{i}}{n_{c}\sum_{i=1}^{n_{c}}y_{i}}-\frac{n_{c}+1}{n_{c}}.$$ (2)

*Disparity index*. We first calculate the weight of collaboration (link) between *a* and *c*_*i*_ as given by Newman [42],

$$w_{ac_{i}}=\sum_{j=1}^{k_{c_{i}}}\frac{1}{l_{j}-1},$$ (3)

where $k_{c_{i}}$ is the number of publications authored by *a* and *c*_*i*_ together, and *l*_*j*_ is the number of co-authors in publication *j*. Then we calculate for *a* the summation of the weights of collaboration (strength),

$$s_{a}=\sum_{i=1}^{n_{c}}w_{ac_{i}}.$$ (4)

Finally, the disparity index is calculated as

$$ϒ\left(a\right)=\sum_{i=1}^{n_{c}}{\left(\frac{w_{ac_{i}}}{s_{a}}\right)}^{2}n_{c}.$$ (5)

We obtain the sample of Gini coefficients for female authors, {*G*_*F*_}, and that for male authors, {*G*_*M*_}. We then can obtain the significance of the difference between the two samples, by performing a Kolmogorov-Smirnov test on the cumulative distribution function curves of the two samples. We perform the same hypothesis test for {*ϒ*_*F*_} and {*ϒ*_*M*_}.

### Simulating total number of distinct co-authors

We simulate the process of accumulating distinct co-authors and then calculate the total number of distinct co-authors. For each author, we calculate the fraction of repeated co-authors, *f*_*r*_. We then generate a list of publications, and record the number of collaborations with each distinct co-author. For each co-author in each publication, we decide if this co-author is a previous co-author with probability *f*_*r*_. If yes, we choose a previous co-author with a probability proportional to the times of collaboration with that co-author, and increase the times of collaboration with that co-author by one. Otherwise, we add a new co-author to the list of co-authors. We do not use equal probability when choosing a previous co-author because this would lead to larger number of distinct co-authors than observed.

Initially, we assign to each author 100 publications, in each of which the author has 5 co-authors. The results show that, for most disciplines, females have significantly more distinct co-authors (*p* < 0.0006, S4A Fig). This is expected since females repeat co-authors less than males do. We next introduce the observed heterogeneity in the team size, by keeping the number of publications at 100 while using team sizes sampled from the author’s publications. S4B Fig shows that in this case the gender difference is no longer significant. Finally, we introduce the heterogeneity in the number of publications, by using the actual number of publications and the number of co-authors in each publication (S4C Fig). Now, females have significantly fewer number of distinct co-authors for most disciplines. These results clearly expose the origins of the results presented in Fig 1 where by controlling for number of publications alone we observed no statistical significant difference between males and females in the number of distinct co-authors.

### Confidence interval for the probability of greater number of co-authors per publication

We consider the probability that publications authored by female authors in our cohort have a larger number of co-authors than publications authored by male authors in our cohort as a function of the career stage of the authors. Since not all the publications are published at the same career stages of the authors, and the size of science teams is increasing with time, we do not consider raw numbers of co-authors but instead standard scores relative to career stages.

Let *n*_*i*_(*y*) denote the number of co-authors of publication *i* from discipline *j* in year *y*, and let *N*_*j*_(*y*) denote the total number of publications published in year *y*. We calculate the standard score of publication *i* in year *y* as

$$z_{i}\left(y\right)=\frac{n_{i}\left(y\right)-\mu_{j}\left(y\right)}{\sigma_{j}\left(y\right)},$$ (6)

where *μ*_*j*_(*y*) is the average number of co-authors per publication from discipline *j* published in year *y*

$$\mu_{j}(y)=\frac{\sum_{k}n_{k}(y)}{N_{j}(y)},$$ (7)

*σ*_*j*_(*y*) is the standard deviation of the number of co-authors per publication published in year *y*

$$\sigma_{j}\left(y\right)=\sqrt{\frac{1}{N_{j}\left(y\right)}\sum_{k}{\left[n_{k}\left(y\right)-\mu_{j}\left(y\right)\right]}^{2}}.$$ (8)

We finally consider $z_{i}^{c}\left(s\right)$, the standard score of publication *i* as a function of the career stage *s* = *y* − *y*_*i*_, where *y*_*i*_ is the year of the first publication of *i*’s author. We then calculate for each career stage *s* the quantity $P[z_{F}^{c}\left(s\right)>z_{M}^{c}\left(s\right)]$, representing the probability that a publication authored by a female author has a standard score higher than that of a publication authored by a male author at the same stage of the career as the female author. We also compute the confidence intervals for these probability values, in the null hypothesis that there is no gender difference in the standard scores:

$$H_{0}:z_{F}\left(t\right)=z_{M}\left(t\right).$$ (9)

We generate the confidence interval valid under this hypothesis using a re-sampling method: The populations of females and males are fixed, the values of all standard scores are also fixed, but values of the standard score are randomly reassigned among publications (this is the same as randomly reassigning the genders to authors). For each random configuration, we compute again the probability $P[z_{F}^{c}\left(s\right)>z_{M}^{c}\left(s\right)]$ and obtain the confidence interval by repeating this procedure 1,000 times.

### Statistical significance of the number of publications with a given team size

To measure the extent to which females have different team sizes than expected, we use the hypergeometric distribution as the null model. We first account for the increasing trend in the team size over years (S5 Fig). For publication *i* with *n*_*i*_ co-authors from discipline *j* in year *y*, we calculate the corrected team size, *ν*_*i*_(*y*), by dividing *n*_*i*_ by the average number of co-authors for all the publications published in year *y*, *μ*_*j*_(*y*),

$$\nu_{i}\left(y\right)=\frac{n_{i}\left(y\right)}{\mu_{j}\left(y\right)},\;\;\mu_{j}\left(y\right)=\frac{\sum_{k}\;n_{k}\left(y\right)}{N_{j}\left(y\right)},$$ (10)

where *N*_*j*_(*y*) is the total number of publications published in year *y*. We then bin the publications according to *ν*(*y*).

For the discipline being considered, suppose there are *N* publications in total, of which *N*_*F*_ are authored by females. Consider a bin *b* in which there are *N*_*b*_ publications. If the females collaborate with teams of different sizes with equal probability, then the expected number of publications by females in *b* is

$$N_{F,b}^{e}=N_{b}\frac{N_{F}}{N}.$$ (11)

Suppose that of the *N*_*b*_ publications in bin *b*, $N_{F,b}^{o}$ are authored by females. The probability of observing $N_{F,b}^{o}$ publications by females given by the hypergeometric distribution is then

P(X=NF,bo)=NFNF,boN-NFNb-NF,boNNb. (12)

The p-value of observing $N_{F,b}^{o}$ is then $P(X\leq N_{F,b}^{o})$. In S6 Fig we plot $\log\frac{N_{F,b}^{o}}{N_{F,b}^{e}}$ for each bin, and shade the regions where the p-value is significant. We use the Bonferroni correction in which the false discovery rate (FDR) is set to be 0.01. We reject the null model if p-value $<\frac{0.01}{m}$, where *m* is the number of bins and thus the number of hypotheses.

## Supporting Information

S1 FigGender differences in the propensity to repeat previous collaboration measured using the Gini coefficient.Distribution of the Gini coefficient of collaboration heterogeneity [33] for females (orange) and males (purple) in the dataset with at least 10 publications. We exclude single-author publications. We obtain *p*-values for the validity of the null hypothesis that the samples were drawn from the same distribution using the Kolmogorov-Smirnov test. For all disciplines, we find $\delta =2\left({\bar{G}}_{F}-{\bar{G}}_{M}\right)/\left({\bar{G}}_{F}+{\bar{G}}_{M}\right)<0$, where ${\bar{G}}_{F}$ and ${\bar{G}}_{M}$ are the average Gini coefficient of the female and male faculty, respectively. Females have Gini coefficients smaller than those of males, suggesting that female faculty have a lower propensity than male faculty to repeat collaborations. Data for this figure are in S6 Data.(EPS)

S2 Fig Gender difference in the propensity to repeat previous co-authors measured using the disparity index. Distribution of the disparity index measuring the repetition of co-authors of females (orange) and males (purple). The *p*-values indicate the significance of the gender difference, obtained with Kolmogorov-Smirnov test. The result is in good agreement with that obtained using the Gini coefficient in S1 Fig. Data for this figure are in S7 Data.
(EPS)

S3 FigCorrelation between Gini coefficient and probability to repeat previous co-authors.Orange (female) and purple (male) lines are linear fits to data, and $R_{F}^{2}$ and $R_{M}^{2}$ are the corresponding coefficient of determination. Data for this figure are in S8 Data.(EPS)

S4 Fig Heterogeneity in the number of publications and team size masks the effect of gender difference in the propensity to repeat co-authors. Survival curves of the simulated total number of distinct co-authors with fixed number of publications and team size (**A**), fixed number of publications and team sizes sampled from real data (**B**), and both number of publications and team sizes from real data (**C**) for female (orange) and male (purple) faculty in all departments (see Materials and Methods). We obtained *p*-values for the validity of the null hypothesis that the samples were drawn from the same distribution using the Kolmogorov-Smirnov test. Statistical significant results with *p* < 0.01/18 ≈ 0.0006 (Bonferroni correction for multiple hypothesis) are shaded grey. When using fixed number of publications and team size, females have significantly more distinct co-authors. However, the gender difference disappears for most disciplines when using fixed number of publications but real team sizes. When we also use number of publications from the real data, females have significantly fewer distinct co-authors, consistent with Fig 1. Data for this figure are in S9 Data.
(EPS)

S5 Fig Growth of average number of co-authors during considered period. Average number of co-authors per publication for females (orange) and males (purple) as a function of publication year. The data are smoothed using a moving averaging method with window size 3. The shaded region indicates the 99% confidence interval obtained with bootstrapping. Data for this figure are in S10 Data.
(EPS)

S6 Fig In molecular biology departments, female faculty work in smaller teams than male faculty. Logarithm of the ratio of observed number of publications authored by females over that expected from a hypergeometric distribution (orange circles). The publications are binned by the number of co-authors corrected for the annual average with a bin size of 0.2. The shaded areas indicate that the observed number is significantly different from expected by the model, using the Bonferroni correction by treating each bin as an independent hypothesis test (see Materials and Methods). The error bars indicate thrice the standard deviation. The black line indicates the ratio of 1.0, and the purple line indicates the average corrected team size. Note that for molecular biology, females have more publications than expected with smaller teams (corrected team size < 1.0) and fewer publications than expected with larger teams (corrected team size > 1.0). Data for this figure are in S11 Data.
(EPS)

S7 Fig Correlation between the average number of co-authors corrected for the annual average versus the fraction of publications authored by female faculty in chemical engineering departments. Publications are grouped by journal. We restricted the publication types to “article”, “letter”, and “note”. The size of the circle is proportional to the logarithm of the number of publications in that journal or sub-discipline. We use journal category in the *ISI Journal Citation Report* as the sub-disciplines. Journals with multiple categories are plotted as concentric rings. The purple line indicates the total average fraction of publications by females for all the publications authored by faculty in chemical engineering in our cohort, *f*_*M*_. The blue line is a weighted linear regression, in which we assign to each journal a weight equal to the number of publications. We only include data points within the range of [0.5*f*_*M*_, 2*f*_*M*_]. Data for this figure are in S4 Data.
(EPS)

S8 Fig Correlation between the average number of co-authors corrected for the annual average versus the fraction of publications authored by female faculty in chemistry departments. See the caption of S7 Fig for details. Data for this figure are in S4 Data.
(EPS)

S9 Fig Correlation between the average number of co-authors corrected for the annual average versus the fraction of publications authored by female faculty in ecology departments. See the caption of S7 Fig for details. Data for this figure are in S4 Data.
(EPS)

S10 Fig Correlation between the average number of co-authors corrected for the annual average versus the fraction of publications authored by female faculty in materials science departments. See the caption of S7 Fig for details. Data for this figure are in S4 Data.
(EPS)

S11 Fig Correlation between the average number of co-authors corrected for the annual average versus the fraction of publications authored by female faculty in psychology departments. See the caption of S7 Fig for details. Data for this figure are in S4 Data.
(EPS)

S1 Table University rankings according to the 2010 edition of the Best Colleges Ranking from US News & World Report [43]. We also show the specialty Graduate School Rankings for Chemical Engineering [44], Chemistry [45], and Ecology [46] when available.
(PDF)

S2 Table University rankings according to the 2010 edition of the Best Colleges Ranking from US News & World Report [43]. We also show the specialty Graduate School Rankings for Materials Science [47], Molecular Biology [48], and Psychology [49] when available.
(PDF)

S3 Table Research topics in molecular biology. We show for each topic the list of most representative words and journals. The topic numbers and words are given by the topic classifying method [35], and the journals are those in which the number of publications is significantly more than expected to occur by chance if drawn from a hypergeometric distribution.
(PDF)

S4 Table The 20 most prolific scientists in our dataset publishing in topic B5 identified as genomics (outlier topic 6 in Table 2). (PDF)

S5 Table The 20 most prolific scientists in our dataset publishing in topic B10 (outlier topic 7 in Table 2). (PDF)

S6 Table The 20 most prolific scientists in our dataset publishing in topic B21 identified as telomere research. (PDF)

S1 Data Data for Fig 1. (XLSX)

S2 Data Data for Fig 2. (XLSX)

S3 Data Data for Fig 3. (XLSX)

S4 Data Data for Fig 4, and S7 Fig through S11 Fig. (XLSX)

S5 Data Data for Fig 5. (XLSX)

S6 Data Data for S1 Fig. (XLSX)

S7 Data Data for S2 Fig. (XLSX)

S8 Data Data for S3 Fig. (XLSX)

S9 Data Data for S4 Fig. (XLSX)

S10 Data Data for S5 Fig. (XLSX)

S11 Data Data for S6 Fig. (XLSX)
