# Supplementary material for: Differences in Collaboration Patterns across Discipline, Career Stage, and Gender
Source: PLoS Biol. 2016 Nov 4;14(11):e1002573. doi: 10.1371/journal.pbio.1002573 (PMC5096717; doi:10.1371/journal.pbio.1002573)
Supplement: S1 Table — We also show the specialty Graduate School Rankings for Chemical Engineering [44], Chemistry [45], and Ecology [46] when available. (PDF) [file pbio.1002573.s012.pdf]

S1 Table. University rankings according to the 2010 edition of the Best Colleges Ranking from US News & World Report.

		Chemical Engineering		Chemistry		Ecology	
Rank	University	Rank	Researchers	Rank	Researchers	Rank	Researchers
1	Harvard University	—	—	5	24	2	11
1	Princeton University	6	22	16	20	8	16
4	Massachusetts Inst of Technology	1	39	1	32	—	—
4	Stanford University	5	20	1	23	7	18
4	Univ Pennsylvania	16	23	20	34	—	—
4	California Inst of Technology	3	10	1	26	—	—
8	Univ Chicago	—	—	—	—	1	20
10	Duke University	—	—	43	27	5	31
12	Northwestern University	16	17	9	30	—	—
14	Johns Hopkins University	23	13	28	21	—	—
15	Cornell University	13	17	9	26	6	25
17	Rice University	23	18	28	22	—	13
17	Emory University	—	—	36	20	—	—
20	Univ Notre Dame	30	20	62	35	—	—
21	Univ California, Berkeley	2	18	1	58	2	43
22	Carnegie Mellon University	16	23	50	28	—	—
24	Univ California, Los Angeles	23	13	12	54	—	—
27	Univ Michigan	13	23	16	48	—	51
35	Georgia Inst of Technology	11	38	26	42	—	—
39	Univ Wisconsin at Madison	6	19	7	46	—	31
39	Univ Illinois at Urbana-Champaign	11	20	7	44	—	68
42	Univ California, Santa Barbara	9	19	26	40	—	—
42	Rensselaer Polytechnic Inst	27	17	74	21	—	—
42	Univ California, Davis	30	27	34	38	—	—
42	Univ Washington	21	17	28	35	—	6
47	Univ Florida	23	22	36	44	—	—
47	Univ Texas at Austin	6	23	12	46	8	16
47	Pennsylvania State University	21	22	16	36	—	58
53	Ohio State University	27	17	28	41	—	—
56	Boston University	—	—	62	22	—	—
58	Univ Georgia	—	—	—	—	10	24
61	Univ Minnesota at Minneapolis St. Paul	3	33	22	42	—	—
61	Purdue University	15	27	22	55	—	—
68	Univ Delaware	10	24	62	32	—	—
77	Univ Colorado	19	22	28	51	—	—
88	North Carolina State University	20	24	50	28	—	—
106	Univ Massachusetts Amherst	30	18	50	27	—	—
