# Supplementary material for: Differences in Collaboration Patterns across Discipline, Career Stage, and Gender
Source: PLoS Biol. 2016 Nov 4;14(11):e1002573. doi: 10.1371/journal.pbio.1002573 (PMC5096717; doi:10.1371/journal.pbio.1002573)
Supplement: S2 Table — We also show the specialty Graduate School Rankings for Materials Science [47], Molecular Biology [48], and Psychology [49] when available. (PDF) [file pbio.1002573.s013.pdf]

S2 Table. University rankings according to the 2010 edition of the Best Colleges Ranking from US News & World Report.

		Materials Science		Molecular Biology		Psychology	
Rank	University	Rank	Researchers	Rank	Researchers	Rank	Researchers
1	Harvard University	—	—	1	44	3	26
1	Princeton University	—	—	8	54	8	30
3	Yale University	—	—	8	38	3	25
4	Massachusetts Inst of Technology	1	25	2	67	—	—
4	Stanford University	5	14	3	24	1	32
4	Univ Pennsylvania	13	20	—	—	—	—
4	California Inst of Technology	12	11	6	15	—	—
10	Duke University	—	24	—	—	—	—
12	Northwestern University	3	35	—	—	—	—
12	Washington University in St. Louis	—	—	—	155	—	—
14	Johns Hopkins University	22	12	8	32	—	—
15	Cornell University	8	17	—	—	—	—
17	Rice University	37	15	—	—	—	—
21	Univ California, Berkeley	5	25	5	41	1	31
22	Carnegie Mellon University	13	19	—	—	—	—
24	Univ California, Los Angeles	22	19	—	—	3	67
27	Univ Michigan	7	30	—	—	3	111
35	Georgia Inst of Technology	8	39	—	—	—	—
39	Univ Wisconsin at Madison	16	20	—	—	8	35
39	Univ Illinois at Urbana-Champaign	2	23	—	—	7	53
42	Univ California, Santa Barbara	4	34	—	—	—	—
42	Rensselaer Polytechnic Inst	19	11	—	—	—	—
42	Univ Washington	26	15	—	—	—	—
47	Univ Florida	8	40	—	—	—	—
47	Univ Texas at Austin	—	—	—	123	—	—
47	Pennsylvania State University	8	24	—	—	—	—
53	Ohio State University	15	24	—	—	—	—
56	Boston University	—	31	—	—	—	—
61	Univ Minnesota at Minneapolis St. Paul	—	—	—	—	8	40
61	Purdue University	16	15	—	—	—	—
68	Univ Delaware	45	14	—	—	—	—
88	North Carolina State University	31	16	—	—	—	—
—	Univ California, San Francisco	—	—	4	50	—	—

Univ California, San Francisco offers only graduate-level courses and thus is not part of the Best Colleges Rankings. We still include it since it is very highly ranked in the specialty of Molecular Biology.
