# Supplementary material for: Differences in Collaboration Patterns across Discipline, Career Stage, and Gender
Source: PLoS Biol. 2016 Nov 4;14(11):e1002573. doi: 10.1371/journal.pbio.1002573 (PMC5096717; doi:10.1371/journal.pbio.1002573)
Supplement: S3 Table — We show for each topic the list of most representative words and journals. The topic numbers and words are given by the topic classifying method [35], and the journals are those in which the number of publications is significantly more than expected to occur by chance if drawn from a hypergeometric distribution. (PDF) [file pbio.1002573.s014.pdf]

S3 Table. Research topics in molecular biology.

Topic	Representative words	Representative journals
B0	cell express activ signal develop	Development, Molecular and Cellular Biology, Cancer Research, Genes & Development, Journal of Immunology
B1	patient increas studi p signific	Journal of Clinical Investigation, Cancer Research, Circulation, Diabetes, Investigative Ophthalmology & Visual Science
B2	protein structur bind dna site	Biochemistry, Nucleic Acids Research, Molecular Cell, Journal of The American Chemical Society, EMBO Journal
B3	use model method data protein	Biophysical Journal, Nucleic Acids Research, Journal of The American Chemical Society, Physical Review Letters, Physical Review B
B4	channel receptor neuron cell activ	Journal of Neuroscience, Neuron, Journal of Neurophysiology, Nature Neuroscience, Journal of Physiology-London
B5	gene mutat sequenc genom chromosom	Nature Genetics, Genetics, Nucleic Acids Research, Genome Research, American Journal of Human Genetics
B6	virus infect cell viral protein	Journal of Virology, Virology, Journal of Immunology, Journal of Experimental Medicine, Nature Medicine
B7	protein membran cell transport vesicl	Journal of Cell Biology, Molecular Biology of The Cell, EMBO Journal, Journal of Cell Science, American Journal of Physiology
B8	male femal behavior sex receptor	Endocrinology, Development, Hormones and Behavior, Developmental Biology, Journal of Comparative Neurology
B9	cell microtubul protein spindl mitot	Journal of Cell Biology, Molecular Biology of The Cell, Current Biology, Genes & Development, Molecular and Cellular Biology
B10	speci sequenc phylogenet group data	Molecular Biology and Evolution, Genetics, American Journal of Botany, Systematic Botany, Molecular Phylogenetics and Evolution
B11	actin cell protein filament myosin	Journal of Cell Biology, Journal of Cell Science, Molecular Biology of The Cell, Current Biology, Neuron
B12	gene methyl histon cell dna	Molecular and Cellular Biology, Genes & Development, Molecular Cell, Genetics, Nature Genetics
B13	protein degrad ubiquitin substrat cell	Molecular Cell, Molecular and Cellular Biology, EMBO Journal, Genes & Development, Journal of Virology

Continued on next page

S3 Table. Continued from previous page

Topic	Representative words	Representative journals
B14	plant express gene protein cell	Plant Cell, Plant Journal, Plant Physiology, Plant Molecular Biology, Molecular Plant-Microbe Interactions
B15	gene v iron bacteria express	Journal of Bacteriology, Molecular Microbiology, Infection and Immunity, Applied and Environmental Microbiology, Biotechnology and Bioengineering
B16	c elsevi right reserv all	Developmental Biology, Bioorganic & Medicinal Chemistry Letters, Tetrahedron Letters, FEBS Letters, Biochemical and Biophysical Research Communications
B17	oxid no activ nitric heme	Biochemistry, Investigative Ophthalmology & Visual Science, Biochemical and Biophysical Research Communications, Archives of Biochemistry and Biophysics, Free Radical Biology and Medicine
B18	beta radic 2 dot center	Biochemistry, Journal of the American Chemical Society, Physical Review B, Physical Review Letters, Inorganic Chemistry
B19	light protein gene express arabidopsi	Plant Cell, Plant Physiology, Plant Journal, Genetics, Planta
B20	proteas inhibitor parasit activ cystein	Biochemistry, Journal of Medicinal Chemistry, Chemistry & Biology, Molecular and Biochemical Parasitology, Bioorganic & Medicinal Chemistry Letters
B21	telomer telomeras rna dna cell	Molecular and Cellular Biology, Genes & Development, Nucleic Acids Research, Molecular Cell, RNA-A Publication of the RNA Society
B22	class cell peptid t molecul	Journal of Immunology, Journal of Experimental Medicine, European Journal of Immunology, Immunity, International Immunology
B23	receptor activ thrombin bind platelet	Blood, Journal of Clinical Investigation, Biochemical Journal, Journal of Pharmacology and Experimental Therapeutics, Molecular Endocrinology
B24	charg lipid cation nmr concentr	Biochemistry, Journal of The American Chemical Society, Biophysical Journal, Langmuir, Journal of General Physiology
B25	cell oxidas neutrophil activ mice	Blood, Journal of Immunology, Journal of Experimental Medicine, Journal of Leukocyte Biology, Calcified Tissue International
B26	tumor dna skin mice adduct	Cancer Research, Molecular Carcinogenesis, Biochemistry, Carcinogenesis, Chemical Research In Toxicology

Continued on next page

S3 Table. Continued from previous page

Topic	Representative words	Representative journals
B27	phage gene genom rate cell	Journal of Bacteriology, Evolution, Molecular Biology and Evolution, Virology, RNA-A Publication of The RNA Society
B28	shock heart heat ventricular cardiac	American Journal of Physiology-Heart and Circulatory Physiology, Circulation Research, Circulation, Journal of Cardiovascular Electrophysiology, Heart Rhythm
B29	anion complex 1 angstrom 2	Journal of The American Chemical Society, Biochemistry, Chemical Communications, Journal of Organic Chemistry, Inorganic Chemistry
B30	mice cholesterol receptor apo cell	Journal of Lipid Research, Journal of Clinical Investigation, Circulation Research, Arteriosclerosis Thrombosis and Vascular Biology, Lipids
B31	zone fluid soil site depth	Development, Journal of Neuroscience, American Journal of Pathology, Geology, Journal of Geophysical Research-Planets
B32	coli e assembl pilus bladder	Infection and Immunity, Journal of Bacteriology, Molecular Microbiology, EMBO Journal, Organic & Biomolecular Chemistry
B33	ant coloni popul speci albican	Genetics, PLOS Biology, Evolution, Molecular Ecology, Insectes Sociaux
B34	surfac cell antibodi use film	Physical Review B, Applied Physics Letters, Langmuir, Journal of Physical Chemistry B, Nature Biotechnology
B35	cell protein signal kinas chemotaxi	Journal of Bacteriology, Molecular Microbiology, Biophysical Journal, Planta, Microbial Ecology
B36	spd lung macrophag cell protein	Journal of Immunology, American Journal of Respiratory Cell and Molecular Biology, Journal of Clinical Investigation, American Journal of Physiology-Lung Cellular and Molecular Physiology, Infection and Immunity
B37	protein aggreg diseas beta prion	Protein Science, Human Molecular Genetics, Annals of Neurology, ACS Chemical Biology, Archives of Neurology
B38	ligand bind structur kringl acid	Biochemistry, Journal of The American Chemical Society, Journal of Biomolecular NMR, Protein Engineering, Proteins-Structure Function and Genetics
B39	resist cell drug efflux mutant	Journal of Bacteriology, Antimicrobial Agents and Chemotherapy, Molecular Microbiology, American Journal of Physiology, Organic & Biomolecular Chemistry

Continued on next page

S3 Table. Continued from previous page

Topic	Representative words	Representative journals
B40	gene cell gut c human	Molecular Microbiology, Eukaryotic Cell, American Journal of Physiology, Journal of Nutrition, Cell Host & Microbe
B41	matrix fiber cell type tissu	Journal of Cell Biology, Journal of Clinical Investigation, Journal of Cell Science, Journal of The Acoustical Society of America, American Journal of Respiratory Cell and Molecular Biology
B42	l cell monocytogen host intracellular	Journal of Bacteriology, Infection and Immunity, Molecular Microbiology, Journal of Immunology, Journal of Experimental Medicine
B43	domain bind type vwf platelet	Blood, Journal of Clinical Investigation, Thrombosis and Haemostasis, Journal of Thrombosis and Haemostasis, Human Gene Therapy
B44	mrna rna express intestin protein	RNA-A Publication of The RNA Society, Journal of Lipid Research, Endocrinology, American Journal of Physiology, Biochemical and Biophysical Research Communications
B45	dna recombin protein meiotic chromosom	Genes & Development, Molecular and Cellular Biology, Genetics, Development, Molecular Cell
B46	express gene cell develop hoxa10	Cancer Research, Development, Developmental Biology, Molecular Endocrinology, Endocrinology
B47	receptor bind cell protein ligand	Bioconjugate Chemistry, Biochemical Journal, Journal of Neurochemistry, Journal of Medicinal Chemistry, Experimental Cell Research
B48	activ insulin acid islet increas	Biochemistry, Diabetes, Circulation Research, Journal of Clinical Investigation, Biochemical Journal
B49	subunit alpha protein gamma beta	Analytical Biochemistry, Archives of Biochemistry and Biophysics, Applied Microbiology and Biotechnology, Molecular Plant-Microbe Interactions, Journal of Phycology
B50	beta alpha termin subunit lh	Molecular Endocrinology, Endocrinology, Molecular and Cellular Endocrinology, Clinical Orthopaedics and Related Research, Bio-Technology
B51	reaction synthesi acid group use	Journal of The American Chemical Society, Biochemistry, Organic Letters, Tetrahedron Letters, Journal of Organic Chemistry
B52	mice diseas cell bone normal	Journal of Clinical Investigation, Blood, Investigative Ophthalmology & Visual Science, Molecular Therapy, Journal of Bone and Mineral Research
B53	protein activ cell kinas inositol	Molecular and Cellular Biology, Biochemical and Biophysical Research Communications, Biochemical Journal, Biotechniques, American Journal of Physiology-Endocrinology and Metabolism

Continued on next page

S3 Table. Continued from previous page

Topic	Representative words	Representative journals
B54	transcript activ promot bind protein	Molecular and Cellular Biology, Genes & Development, Molecular Cell, EMBO Journal, Journal of Virology
B55	cell protein assembl flagellar cilia	Journal of Cell Biology, Development, Cell Motility and The Cytoskeleton, Current Biology, Genetics
B56	cell oscil neuron period cycl	Journal of Neuroscience, Neuron, Journal of Neurophysiology, Nature Neuroscience, PLOS One
B57	reductas degrad protein cell j	Archives of Biochemistry and Biophysics, Circulation, American Journal of Medical Genetics, Calcified Tissue International, Protein Expression and Purification
B58	mitochondri cell protein death mitochondria	Molecular and Cellular Biology, Journal of Clinical Investigation, Circulation Research, Archives of Biochemistry and Biophysics, Current Genetics
B59	replic cell gene dna protein	Journal of Bacteriology, Molecular Microbiology, Genes & Development, Molecular Cell, Genetics
B60	toxin alpha nakatpas express cell	Developmental Biology, FEBS Letters, Memorias Do Instituto Oswaldo Cruz, Insect Biochemistry and Molecular Biology, European Journal of Biochemistry
B61	gene express protein cell 1433	Plant Physiology, Plant Molecular Biology, Plant Cell, Plant Journal, Maydica
B62	neuron express gene olfactori drosophila	Development, Neuron, Journal of Neuroscience, Genes & Development, Developmental Biology
B63	m tuberculosi infect immun secret	Infection and Immunity, Molecular Microbiology, Journal of Experimental Medicine, PLOS Pathogens, Structure
B64	protein coli respons gene stress	Journal of Bacteriology, Genes & Development, Molecular Microbiology, Molecular Cell, Molecular Biology of The Cell
B65	gene element boundari express domain	Development, Genes & Development, Molecular and Cellular Biology, Genetics, Nucleic Acids Research
B66	protein activ kinas signal inhibit	Chemistry & Biology, Cell Calcium, Science Signaling, Journal of Experimental Biology, Mutation Research
B67	activ kinas enzym acid phosphoryl	Biochemistry, Journal of Bacteriology, Biotechnology and Bioengineering, Applied Microbiology and Biotechnology, Archives of Biochemistry and Biophysics
B68	respons call pattern select differ	Journal of Neuroscience, Journal of Neurophysiology, Journal of The Acoustical Society of America, Journal of Molecular Evolution, Hearing Research
