# Supplementary material for: Differences in Collaboration Patterns across Discipline, Career Stage, and Gender
Source: PLoS Biol. 2016 Nov 4;14(11):e1002573. doi: 10.1371/journal.pbio.1002573 (PMC5096717; doi:10.1371/journal.pbio.1002573)
Supplement: S4 Table — (PDF) [file pbio.1002573.s015.pdf]

S4 Table. The 20 most prolific scientists in our dataset publishing in topic B5 identified as genomics (outlier topic 6 in Table 2 of main text).

Name	Publications in topic	Total publications	Gender
Lander ES	196	334	M
Vogelstein B	187	448	M
Chakravarti A	99	277	M
Boeke JD	86	220	M
Housman DE	85	213	M
Wilson RK	84	125	M
Botstein D	80	391	M
Kazazian HH	79	320	M
Permutt MA	77	204	M
Page DC	70	177	M
Kruglyak L	67	116	M
Walbot V	62	188	F
Zack DJ	61	149	M
Feinberg AP	60	149	M
Tilghman SM	52	131	F
Nathans J	51	144	M
Silver LM	43	150	M
Germino GG	39	86	M
Burge CB	33	51	M
Landweber LF	30	90	F
