# Supplementary material for: Differences in Collaboration Patterns across Discipline, Career Stage, and Gender
Source: PLoS Biol. 2016 Nov 4;14(11):e1002573. doi: 10.1371/journal.pbio.1002573 (PMC5096717; doi:10.1371/journal.pbio.1002573)
Supplement: S5 Table — (PDF) [file pbio.1002573.s016.pdf]

S5 Table. The 20 most prolific scientists in our dataset publishing in topic B10 (outlier topic 7 in Table 2 of main text).

Name	Publications in topic	Total publications	Gender
Jansen RK	114	148	M
Hillis DM	49	143	M
Gutell RR	48	96	M
Andolfatto P	22	37	M
Warnow T	21	49	F
Lander ES	16	334	M
Wilson RK	14	125	M
Garcia BA	14	91	M
Hoekstra HE	13	38	F
Landweber LF	12	90	F
Shankland M	12	42	M
Irish VF	11	54	F
Dellaporta SL	10	51	M
Barrick JE	10	43	M
Silver LM	9	150	M
Matz MV	9	34	M
Gordon JI	8	396	M
Deng XW	8	200	M
Weissman JS	8	196	M
Bartel DP	8	121	M
