# Supplementary material for: Differences in Collaboration Patterns across Discipline, Career Stage, and Gender
Source: PLoS Biol. 2016 Nov 4;14(11):e1002573. doi: 10.1371/journal.pbio.1002573 (PMC5096717; doi:10.1371/journal.pbio.1002573)
Supplement: S6 Table — (PDF) [file pbio.1002573.s017.pdf]

S6 Table. The 20 most prolific scientists in our dataset publishing in topic B21 identified as telomere research.

Name	Publications in topic	Total publications	Gender
Blackburn EH	89	177	F
Zakian VA	59	109	F
Greider CW	58	86	F
Collins K	42	64	F
Campbell JL	27	117	F
Pardue ML	24	117	F
Weinberg RA	23	346	M
Boeke JD	20	220	M
Lambowitz AM	19	174	M
Bartel DP	19	121	M
Hanawalt PC	15	262	M
Sharp PA	11	396	M
Doudna JA	11	115	F
Altman S	10	170	M
Hemann MT	10	25	M
Kazazian HH	9	320	M
Bustamante C	9	205	M
Landweber LF	9	90	F
Paull TT	9	37	F
Vogelstein B	8	448	M
